# The Effects of Vitamin D Supplementation on Hepatic Dysfunction, Vitamin D Status, and Glycemic Control in Children and Adolescents with Vitamin D Deficiency and Either Type 1 or Type 2 Diabetes Mellitus

**DOI:** 10.1371/journal.pone.0099646

**Published:** 2014-06-11

**Authors:** Benjamin Udoka Nwosu, Louise Maranda

**Affiliations:** 1 Division of Endocrinology, Department of Pediatrics, University of Massachusetts Medical School, Worcester, Massachusetts, United States of America; 2 Department of Quantitative Health Sciences, University of Massachusetts Medical School, Worcester, Massachusetts, United States of America; University of Catanzaro Magna Graecia, Italy

## Abstract

**Background:**

The effects of vitamin D supplementation on mild hepatic dysfunction and glycemic control are unclear in children and adolescents with either type 1 (T1D) or type 2 diabetes (T2D).

**Hypothesis:**

Vitamin D supplementation will improve hepatic dysfunction and glycemic control.

**Aim:**

To determine the effect of vitamin D supplementation on alanine transaminase (ALT), hemoglobin A1c (HbA1c), and serum 25-hydroxyvitamin D [25(OH)D] concentration.

**Subjects and Methods:**

A retrospective study of 131 subjects with either T1D (n = 88∶46 females, 42 males), or T2D (n = 43∶26 females, 17 males) of ages 3–18 years between 2007–2013. All subjects had (1) a diagnosis of diabetes for >12 mo, (2) received vitamin D supplementation for the management of vitamin D deficiency (3) had baseline and subsequent simultaneous measurements of HbA1c, ALT, and 25(OH)D. Vitamin D deficiency was defined as 25(OH)D concentration of <50 nmol/L (20 ng/mL).

**Results:**

At baseline, vitamin D deficiency occurred in 72.1% of patients with T2D and in 37.5% of T1D patients (p<0.001). Patients with T2D had significantly higher values for BMI SDS (p<0.001), alanine transaminase (ALT) (p = 0.001), but lower 25(OH)D (p<0.001), and no difference in HbA1c (p = 0.94), and total daily dose (TDD) of insulin per kg body weight (p = 0.48) as compared to T1D patients. After 3 months of vitamin D supplementation, there was a significant increase in 25(OH)D in both T2D (p = 0.015), and T1D patients (p<0.001); significant reduction in BMI SDS (p = 0.015) and ALT (p = 0.012) in T2D, but not in T1D. There was a clinically-significant decrease in HbA1c in T2D from 8.5±2.9% at baseline to 7.7±2.5 at 3 mo, but not in T1D, 8.5±1.2 to 8.53±1.1%.

**Conclusions:**

Vitamin D supplementation in subjects with T2D was associated with statistically significant decreases in BMI SDS, ALT, and a clinically-significant decrease in HbA1c.

## Introduction

The management of diabetes mellitus remains an enigma even though its symptoms were described more than 2000 years ago. This is because the central therapeutic goal of diabetes management, euglycemia, is influenced by complex physiologic and pathologic processes, some of which are clearly understood, while others are less clear. Suboptimal glycemic control is a recognized risk factor for acute and chronic complications of diabetes including microvascular and macrovascular diseases [1]–[3]. However, the role of comorbid states such as nonalcoholic fatty liver disease (NAFLD) and vitamin D deficiency on glycemic control in children and adolescents has not been fully elucidated. Poor glycemic control remains a growing problem in patients with type 1 (T1D) or type 2 diabetes (T2D) [4] despite improvements in insulin formulation, delivery and adjunctive therapies [5]. A recent national study reported that a high proportion of youth with diabetes had elevated hemoglobin A1c (HbA1c) values, with 17% of patients with TIDM and 27% of those with T2DM showing significantly poor control, defined as HbA1c ≥9.5% [4].

NAFLD is the most common form of liver dysfunction in children [6], and the leading cause of elevated liver enzymes in obese youth [7]. Even though its prevalence is rising in parallel with the prevalence of childhood obesity [8], its role in poor glycemic control in diabetes is unknown. NAFLD represents a spectrum of conditions characterized by macrovesicular hepatic steatosis and little or no exposure to alcohol [8]. The hepatic pathology encompasses a range from isolated fatty infiltration to steatohepatitis, advanced fibrosis, and cirrhosis [6].

NAFLD has been strongly associated with vitamin D deficiency [9], [10], IR [11], prediabetes [12], and T2D [10], [13]–[17]. Several studies have reported associations between liver dysfunction and low vitamin D levels [9], [10], [18]–[20] on one hand, and liver dysfunction and poor glycemic control on the other [21]. Our group showed that hepatic dysfunction, characterized by elevated transaminases, was associated with vitamin D deficiency and poor glycemic control in children and adolescents with T2D, but not T1D [22]. This has led to the hypothesis that mild hepatic dysfunction could impair the conversion of vitamin D to 25-hydroxyvitamin D [25(OH)D], and consequently result in vitamin D deficiency. The resultant vitamin D deficiency is believed to impair insulin sensitivity [23].

Vitamin D deficiency is prevalent in children and adolescents with diabetes mellitus [22], [24], [25]. The etiology of this vitamin D deficiency is often attributed to the lack of sun exposure, poor intake of vitamin D-containing foods such as salmon, or due to either volumetric dilution [26], or sequestration of vitamin D in fat depots [27]. However, a crucial step in the metabolism of vitamin D, the hydroxylation of vitamin D at the 25 position, occurs in the liver. The role of NAFLD on this critical step in vitamin D metabolism in children and adolescents with diabetes has not been fully studied [22]. Equally, the effects of the resultant vitamin D deficiency on glycemic control in these patients are unclear. A study in healthy, non-diabetic adults with normal glucose tolerance using the hyperglycemic clamp technique showed a positive correlation of 25(OH)D level with insulin sensitivity [23]. Extrapolation of the data suggested that increasing the serum concentration of 25(OH)D from 25–80 nmol/L would increase insulin sensitivity by 60% [23], indicating that perhaps vitamin D supplementation offers promise as an adjunctive therapy for those with diabetes mellitus [28]. Recent studies in non-diabetic children and adolescents showed that low levels of 25(OH)D were associated with increasing insulin resistance in patients at risk for diabetes [29], while a 12-week study involving children, adolescents and young adults with T1D showed improved HbA1c level following a combined vitamin D and calcium supplementation protocol [30].

Therefore, to achieve the elusive goal of glycemic control in diabetes, euglycemia, it is necessary to evaluate the role of comorbid states such as hepatic dysfunction and vitamin D deficiency on glycemic control. Even though our group showed that hepatic dysfunction was associated with vitamin D deficiency and poor glycemic control in patients with T2D and not in those with T1D, the effects of vitamin D supplementation on the coexistence of hepatic dysfunction, vitamin D deficiency, and poor glycemic control have not been adequately studied in children and adolescents with either T1D or T2D. We hypothesize that vitamin D supplementation would improve vitamin D status, reduce hepatic dysfunction, and in turn lead to improved glycemic control in children and adolescents with diabetes mellitus. The study’s aim was to determine the effect of vitamin D supplementation on ALT, HbA1c, and 25(OH)D in patients with vitamin D deficiency and either T1D or T2D.

## Subjects and Methods

### Ethics Statement

The study protocol was approved by the University of Massachusetts Institutional Review Board. All patient records and information were anonymized and de-identified prior to analysis.

### Subjects

The clinical records of children and adolescents of ages 3–18 years who were treated for T1D and T2D at the Children’s Medical Center of the University of Massachusetts between 2007 and 2013 were reviewed. Subjects were included if they had a diagnosis of diabetes for >12 mo, had HbA1c and 25(OH)D obtained simultaneously, and were on no vitamin D or calcium supplements. Subjects were excluded if they had vitamin D or calcium supplementation at baseline, or the disorders of calcium metabolism, hemoglobinopathies, or malabsorption syndrome such as celiac disease. Eighty-eight subjects with T1D (46 females, 42 males) and 43 subjects with T2D (26 females and 17 males) fulfilled these criteria (Table 1). The diagnosis of T2D was based on fasting blood glucose of ≥7 mmol/L (126 mg/dL), and/or 2-hour postprandial glucose of ≥11.1 mmol/L (200 mg/dL), and/or random blood glucose of ≥11.1 mmol/L (200 mg/dL) with symptoms of polyuria and/or polydipsia. All patients with T2D had negative results for T1D-associated antibodies (insulin, islet cell, glutamic acid decarboxylase, and insulinoma associated-2). Because 25(OH)D level could vary with sunlight exposure, we categorized each subject’s visit according to the seasons as follows: fall (September 22–December 21), winter (December 22–March 21), spring (March 22– June 21), and summer (June 22-September 21) [31]. All subjects with T1D received insulin only. Of the 43 patients with T2D, 17 patients received a combination of insulin and metformin, 15 patients received metformin only, while 8 patients received insulin monotherapy.

**Table 1:** Comparison of the characteristics of patients with type 1 diabetes vs. type 2 diabetes.

Parameters	Type 1 Diabetes (n = 88)	Type 2 Diabetes (n = 43)	*p*
Age (years)	12.6±3.6	16.2±2.2	**<0.001**
Sex: males (%)	42 (47.7%)	17 (39.5%)	0.38*
Race: white (Non-Hispanic) (%)	73 (83.0%)	23 (53.5%)	**0.001** *
Height SDS (baseline)	0.04±1.1	0.2±1.2	0.57
Height SDS (3mo)	0.04±1.1	0.04±1.2	1.0
Weight SDS (baseline)	0.69±1.0	2.3±0.9	**<0.001**
Weight SDS (3mo)	0.71±1.0	2.3±1.0	**<0.001**
Body Mass Index SDS (baseline)	0.80±0.9	2.2±0.6	**<0.001**
Body Mass Index SDS (3mo)	0.81±0.8	2.14±0.7	**<0.001**
Alanine Transaminase (U/L)	19.2±6.2	48.3±47.4	**0.001**
25(OH)D level (nmol/L)	53.7±14.6	41.2±15.3	<0.001
25(OH)D level (nmol/L) in Whites	54.0±14.3	42.0±15.9	0.004
Percentage Vitamin D Deficiency[25(OH)D <50 nmol/L] (%)	33 (37.5%)	31 (72.1%)	**<0.001** *
Hemoglobin A1c (%)	8.5±1.2	8.5±2.9	0.94
Seasons (Summer-Fall)	50/88 (56.8%)	17/43 (39.5%)	*0.063
Duration of Diabetes Mellitus	2.9±1.2	2.03±1.29	0.001

* = p values by Chi Square for proportions; SDS = standard deviation score; 25(OH)D = 25hydroxyvitamin D.

### The Procedure for Vitamin D Therapy

The rationale for the vitamin D supplementation in these patients was based on recent reports of high prevalence of vitamin D deficiency in children and adolescents with either T1D or T2D [22], [24], [25]. It is the standard practice at the Children’s Medical Center to monitor the vitamin D status of children and adolescents with diabetes mellitus during patients’ yearly blood draw for the evaluation of complications of diabetes mellitus, or at one of the patients’ quarterly visits for routine diabetes care. Patients with serum 25(OH)D concentration of <50 nmol/L were started on vitamin D supplementation using either ergocalciferol or cholecalciferol with the aim of raising the 25(OH)D level to >75 nmol/L.

All participants received either ergocalciferol or cholecalciferol for a period ranging from 8 weeks for those receiving 7000 IU/day, through 12–16 weeks for subjects receiving 400–4000 IU/day as shown in Table 2. The dose of vitamin D was based on patient’s age and weight according to reports showing that 25(OH)D response to vitamin D supplementation is directly related to dose and body size [32].

**Table 2:** Summary of Patients’ Vitamin D Treatment Plan.

Treatment Plan	Number of Subjects	
Dose of Vitamin D (IU/day)	Type 1 diabetes	Type 2 diabetes
<1000	3	1
1000–2000	81	26
4000	4	6
7000	0	10

### Anthropometry

Height was measured to the nearest 0.1 cm using a wall-mounted stadiometer (Holtain Ltd, Crymych, Dyfed, UK). Weight was measured to the nearest 0.1 kg using an upright scale. Body mass index (BMI) was derived using the formula weight/height^2^ (kg/m^2^), and expressed as SDS for age and gender based on National Center for Health Statistics (NCHS) data [33].

### Biochemical Studies

Data were collected from quarterly HbA1c estimation during clinic visits. Data on serum concentrations of alanine transaminase (ALT) were obtained simultaneously with HbA1c during routine clinic visit before and after vitamin D supplementation in both T1D and T2D patients. Specifically, ALT, 25(OH)D and other laboratory studies were estimated annually or bi-annually, as part of a routine screening protocol for complications of diabetes mellitus. Patients with vitamin D deficiency were started on vitamin D supplementation, and had a follow up laboratory evaluation for 25(OH)D level at their follow up quarterly visit. Vitamin D supplementation was either discontinued if vitamin D levels became normal upon retesting, or supplementation was continued for another cycle of treatment to attain normal vitamin D status. Data were collected on HbA1c levels obtained 3 months prior to the date of vitamin D supplementation, at the time of the initiation of vitamin D supplementation, and at +3mo, +6mo, and +9mo for the evaluation of the temporal effect of vitamin D supplementation on HbA1c levels.

### Assays

Hemoglobin A1c was measured by DCA 2000+ Analyzer (Bayer, Inc., Tarrytown, NY, USA) based on Diabetes Control and Complications Trial standards [34]. Serum levels of 25(OH)D were analyzed using 25-hydroxy chemiluminescent immunoassay (DiaSorin Liaison; Stillwater, Minnesota), which has a 100% cross-reactivity with both metabolites of 25(OH)D namely, 25(OH)D_2_ and 25(OH)D_3_ and thus measures total serum 25(OH)D content. Its functional sensitivity is 10 nmol/L (4 ng/mL), and its intra- and inter-assay coefficients of variation are 5% and 8.2% respectively.

### Vitamin D Status

Vitamin D sufficiency was defined as a 25(OH)D concentration ≥30 ng/mL (75 nmol/L); vitamin D insufficiency as 25(OH)D of 20 to 29.9 ng/mL (50 to 75 nmol/L); and vitamin D deficiency as 25(OH)D level <20 ng/mL (50 nmol/L) according to the Endocrine Society criteria [35].

### Statistical Analyses

Statistical analyses were performed using the Predictive Analytics SoftWare v.21 (IBM Corporation, Armonk, NY). Means, standard deviations, and percentages were calculated for descriptive summary statistics. The values for ALT were log transformed before analysis to approximate normal distributions. Comparisons between independent means were made using two-tailed Student’s t test and ANOVA. Non-independent measurements over time were compared using paired t test and repeated-measures ANOVA. Proportions were compared using Chi square test; Fisher’s exact test was used where marginal totals were <5. Height, weight, and BMI were expressed as a standard deviation score (SDS).

#### Power analysis

The study’s primary goal was to detect significant changes in HbA1c in subjects with either T1D or T2D during the 3 mo period of vitamin D supplementation, and secondarily to determine if there was a persistence of the changes in HbA1c over a 9 mo period. The small effect size of +0.03% change in HbA1c during the 3 mo of vitamin D supplementation in the 88 patients with T1D yielded a power of 7.5%, while the clinically-significant effect size of −0.6% in 37 subjects with T2D who had pre- and post-treatment values for HbA1c yielded a power of 60.4% at a *p* value of 0.024. Further extrapolation showed that 1,982 subjects with T1D would be needed to achieve a power of 60.4%; while 3,175 patients with T1D, and 58 patients with T2D would be needed to achieve a power of 80%.

## Results

One hundred and thirty one subjects of ages 3–18 years (male 13.8±3.4 years, female 13.7±3.8, p = 0.80) were studied. This consisted of 88 subjects (42 males, 46 females) with T1D of mean age 12.6±3.6 years (males 13.1±3.5; females 12.1±3.6, p = 0.23); and 43 subjects (17 males, 26 females) with T2D of mean age 16.2±2.2 years (males 15.8±2.2; females 16.4±2.2, p = 0.36) (Table 1).

At baseline, 72.1% of patients with T2D had vitamin D deficiency compared to 37.5% of T1D patients (p<0.001). Patients with T2D had significantly higher values than those with T1D for BMI SDS (2.2±0.6 vs. 0.80±0.9, p<0.001), ALT (48.3±47.4 vs. 19.2±6.2, p = 0.001), but lower mean 25(OH)D level (41.2 nmol/L ±15.3 vs. 53.7±14.6, p<0.001), and no difference in HbA1c (8.5±2.9% vs. 8.5±1.2, p = 0.94) and total daily dose (TDD) of insulin per kg body weight (0.85 units/kg ±0.4 vs. 0.88±0.3, p = 0.48) (Table 3).

**Table 3:** Comparison of the changes in the doses of insulin and metformin during the period of vitamin D supplementation.

Parameters	Type I Diabetes (n = 88)		*p*	Type 2 Diabetes (n = 43)		*p*
Time (months)	0	3		0	3	
TDD of insulin (units)	48.0±26.4	49.4±26.7	**0.008**	75.6±36.1	73.1±35.8	0.23
TDD of insulin per kgbody weight (units/kg)	0.90±0.3	0.91±0.3	0.54	0.85±0.4	0.80±0.41	0.067
TDD of metformin (mg)	N/A	N/A	N/A	1105.0±687.4	122.3±670.0	0.052
TDD of metformin per kgbody weight (mg/kg)	N/A	N/A	N/A	10.71±7.5	12.3±8.0	0.060

TDD = total daily dose.

**Figure 1:**
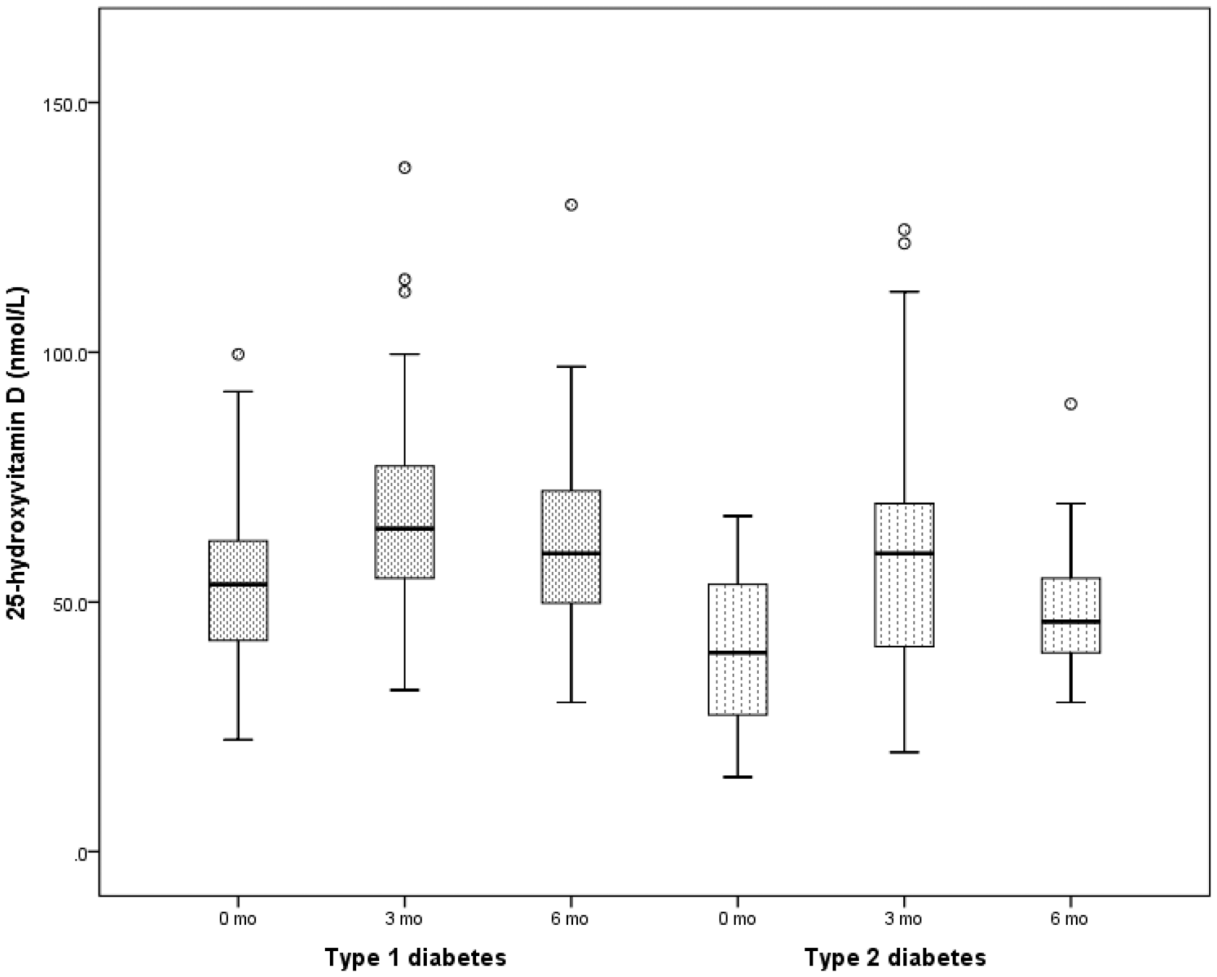
Graph showing a rise in the serum concentrations of 25-hydroxyvitamin D [25(OH)D] during vitamin D supplementation in patients with either type 1 diabetes or type 2 diabetes. In patients with type 1 diabetes, 25(OH)D rose from a baseline of 53.3±14.6 nmol/L to a peak of 67.6±20.6 at 3 mo, and then decreased to 62.4±18.4 (ANOVA p<0.001). In patients with type 2 diabetes 25(OH)D rose from a baseline value of 41.8±15.0 to a peak of 60.7±27.7 at 3 months and then decreased to 49.2±16.6 (ANOVA p = 0.011).

**Figure 2:**
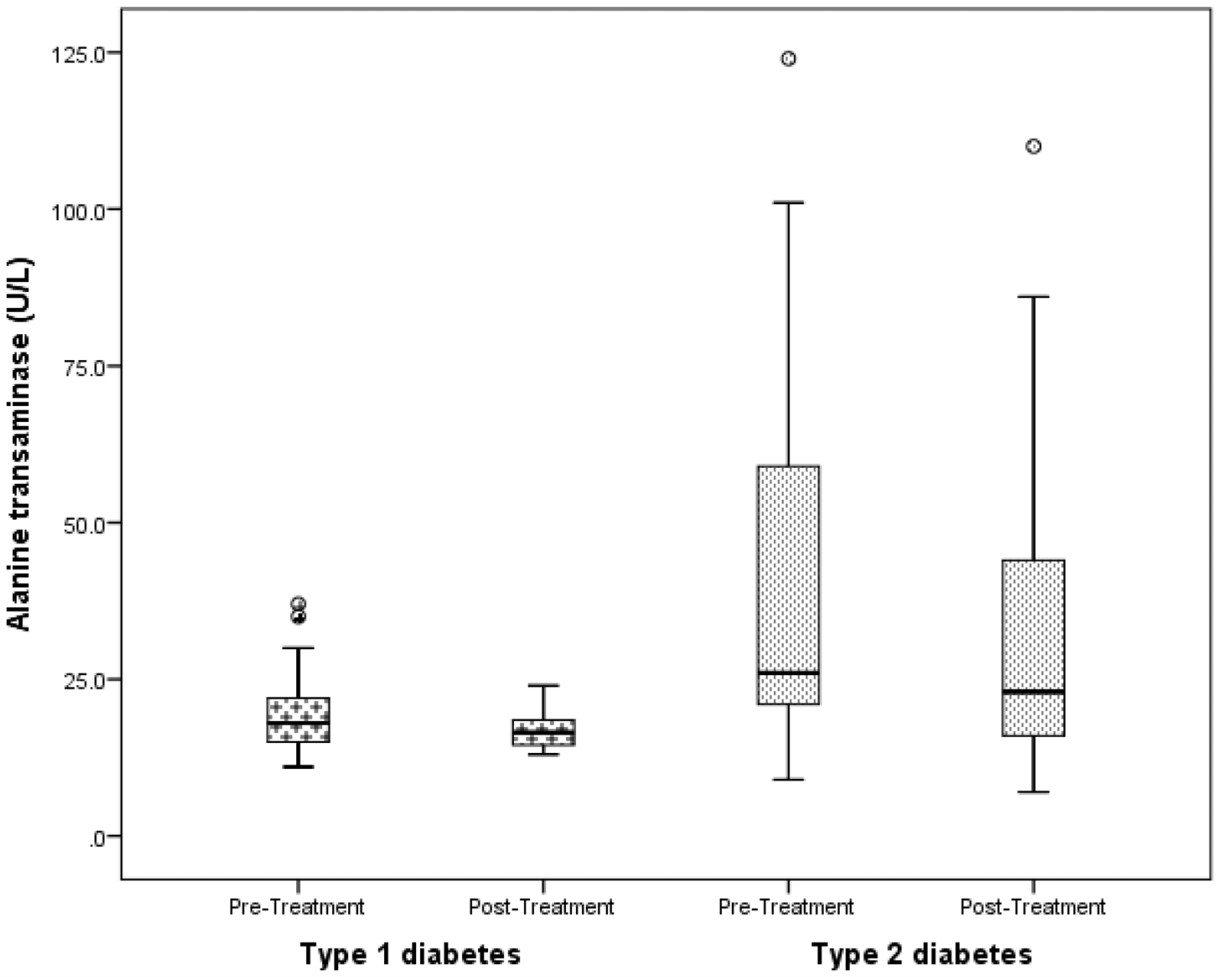
Bar graph showing the temporal change in the levels of alanine transaminases (ALT) following vitamin D supplementation. ALT decreased from 18.0±6.0 U/L to 15.7±2.0 (p = 0.46) in patients with type 1 diabetes; and from 50.7±50.9 to 35.0±31.0 (p = 0.012) in those with type 2 diabetes.

At 3 months, following vitamin D supplementation, 25(OH)D increased significantly in patients with T1D from a baseline of 53.3±14.6 nmol/L to a peak of 67.6±20.6 at 3 mo, and then decreased to 62.4±18.4 nmol/L (ANOVA p<0.001) (Figure 1). Similarly, in patients with T2D, 25(OH)D rose significantly from a baseline value of 41.8±15.0 nmol/L to a peak of 60.7±27.7 at 3 months and then subsequently decreased to 49.2±16.6 nmol/L (ANOVA p<0.011). There was neither a change in the TDD of insulin per kg body weight in either T1D or T2D, nor in the TDD of metformin per kg body weight in T2D patients (Table 3). Following vitamin D supplementation, there was a significant decrease in ALT in T2D (50.7±50.9 U/L to 35.0±31.0, p = 0.012), but not in T1D (18.0±6.0 U/L to 15.7±2.0 (p = 0.46) (Figure 2).

An analysis of the anthropometric changes in the subjects during the 3-month period of vitamin D supplementation showed no differences in weight SDS in either the T1D (0.70±1.0 to 0.71±1.0, p = 0.31) or T2D (2.31±0.96 to 2.26±0.96, p = 0.13) cohort. In contrast, there was a significant decrease in BMI SDS in patients with T2D (2.21±0.62 to 2.14±0.66, p = 0.015), but not in patients with T1D (0.80±0.88 to 0.81±0.84, p = 0.61).

**Figure 3:**
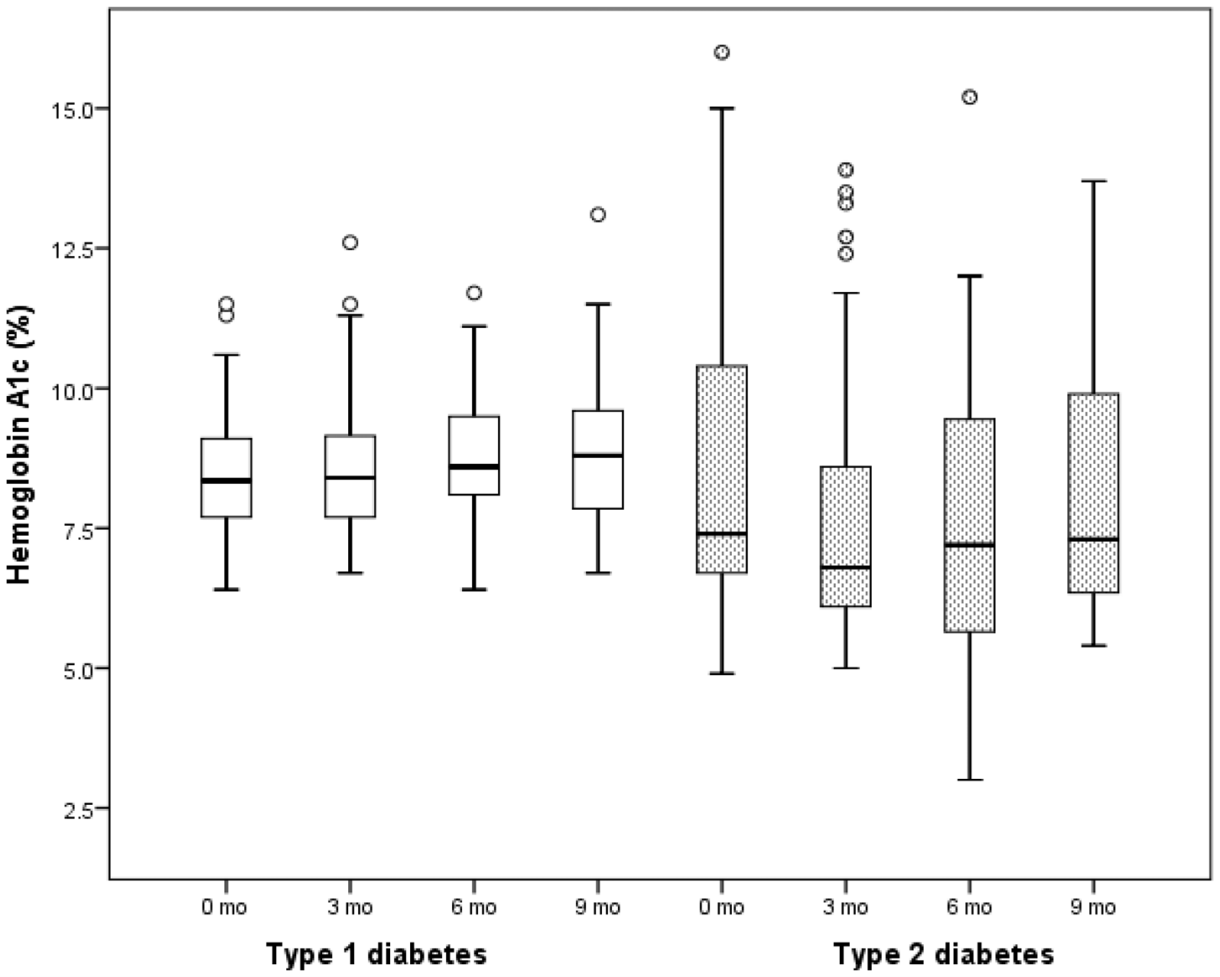
A 9-month analysis of the changes in hemoglobin A1c (HbA1c) level following a three-month vitamin D supplementation in patients with either type 1 diabetes or type 2 diabetes. There was a statistically-significant, but clinically non-significant rise in HbA1c level over 9 months in T1D (8.5±1.2 through 8.9±1.2%, ANOVA p = 0.001). In T2D, there was a clinically significant decrease in HbA1c [36] in the first 3 months (during the period of vitamin D supplementation) from 8.5±2.9% at baseline through 7.7±2.5% at 3 mo, followed by a slow return to pretreatment values of 8.2±2.3% at 9 mo. There was however, no statistical significant difference in HbA1c between the time points (ANOVA p = 0.52).

A comparison of changes in HbA1c level over a period of 9 mo from the time of the initiation of vitamin D supplementation in subjects with either T1D or T2D showed a significant difference in HbA1c between the subjects with T1D and those with T2D (ANOVA p = 0.011). A focused analysis of the changes in HbA1c over the 9-month period showed a statistically significant, but clinically non-significant increase in HbA1c from baseline to 9 mo (p = 0.001) in the T1D cohort, while the patients with T2D had no change in A1c (p = 0.52). A closer examination of the HbA1c trends during the 9 mo showed that in patients with T1D, HbA1c levels increased consistently over time from 8.5±1.2 through 8.9±1.2% (ANOVA p = 0.001), while in patients with T2D, the HbA1c value decreased at the beginning of the supplementation (8.5±2.9% at baseline through 7.7±2.5% at 3 mo) but slowly returned toward the baseline value of 8.2±2.3% at the end of 9 months (Figure 3). To investigate whether pre-treatment excursions in HbA1c accounted for the changes in HbA1c described above for T1D and T2D, we compared the mean of the HbA1c levels drawn 3 mo before the start of vitamin D supplementation to the mean of the baseline HbA1c drawn just before the start of vitamin D therapy. There were no significant differences in HbA1c values in T1D (8.4±1.3% vs. 8.5±1.2, p = 0.57), and in T2D (8.9±3.3 vs. 8.5±2.9, p = 0.26).

## Discussion

This study evaluated the effects of vitamin D supplementation on hepatic dysfunction and glycemic control in children and adolescents with vitamin D deficiency and either T1D or T2D. At baseline, a greater proportion of subjects with T2D were obese, and had both vitamin D deficiency and elevated ALT levels compared to those with T1D. The 3-mo period of vitamin D supplementation in subjects with T2D was associated with significant reduction in BMI SDS, ALT, and a clinically-significant decrease in HbA1c which was followed by a gradual return toward pretreatment HbA1c values. These reductions in BMI SDS, ALT, and HbA1c [36] in T2D patients were not associated with significant changes in the doses of either insulin or metformin. These data and our earlier report [22] suggest that vitamin D deficiency may be associated with mild hepatic dysfunction which could in turn impair glycemic control in patients with T2D. The normalization of serum vitamin D concentration through vitamin D supplementation appeared to transiently improve glycemic control in patients with T2D.

Our data are in agreement with studies showing a higher prevalence of vitamin D deficiency in pediatric patients with T2D compared to those with T1D [37]. The clinically-significant reduction in HbA1c in our T2D cohort (Figure 2) is consistent with reports of significant associations between vitamin D and glycemic control in T2D [38], as well as earlier reports linking vitamin D deficiency with glucose intolerance [39], hyperinsulinemia [40], obesity [41], and T2D. This clinically-significant reduction in HbA1c in subjects with T2D is similar to the findings of a meta-analysis [36] on the effect of exercise on HbA1c in patients with T2D, which reported a decrease in HbA1c of 0.6%, with associated significant reductions in both visceral and subcutaneous adipose tissues in the study patients.

### Vitamin D Deficiency and Type 1 Diabetes

Though vitamin D deficiency has been associated with increased risk of developing T1D [42], [43], the relationship between 25(OH)D and glycemic control in patients with T1D is not fully understood. Our previous study [22] found no significant relationship between 25(OH)D and HbA1c in patients with T1D, while this study found a statistically significant, but clinically non-significant increase in HbA1c value following vitamin D supplementation. These results differ from the conclusions of a study in children, adolescents, and young adults with T1D which found a significant reduction in HbA1c level following a supplementation regimen consisting of both vitamin D and calcium [30]. It is possible that vitamin D alone has no significant role on improving glycemic control in patients with T1D.

### Vitamin D Deficiency and Type 2 Diabetes

There is no consensus on the effect of vitamin D supplementation on HbA1c level in T2D. Even though previous studies [22], [44], [45] had reported strong inverse relationship between 25(OH)D and HbA1c, this association alone is not enough to demonstrate a cause and effect relationship. Evidence from two randomized controlled trials (RCT) suggests that vitamin D supplementation effectively reduces insulin resistance (IR) [46], [47], but three smaller RCTs [48]–[50] of vitamin D supplementation in patients with T2D reported no change in HbA1c following vitamin D supplementation. A closer examination of these three RCTs showed several limitations: (1) all three studies were underpowered (only 16–20 participants per arm), (2) only one study [50] was designed for glycemic outcomes, but it recruited only about half of the planned cohort, and studied patients with normal 25(OH)D levels; (3) only one study [48] reported baseline use of diabetes medications; however, in that study metformin use rate was much higher in the vitamin D arm; (4) changes in diabetes medications were not reported and were not taken into account in the analyses; and (5) all three trials used large infrequent doses of vitamin D. Two recent randomized studies reported improved glycemic control in patients with T2D who either received vitamin D alone [51] or vitamin D fortified yogurt drink [52] while another study reported decreased levels of inflammatory markers following vitamin D supplementation in patients with T2D [53]. However, a Japanese study reported no change in HbA1c following 2000 IU per day of vitamin D_3_ with calcium for 24 weeks in patients with T2D [54]. Thus, the effects of vitamin D supplementation on glycemic control in patients with T2D are unclear in adults, and more so in children and adolescents where there are only a few published studies.

There was a significant decrease in BMI SDS in patients with T2D following vitamin D supplementation. The high prevalence of obesity and vitamin D deficiency at baseline in subjects with T2D is consistent with a report that characterized vitamin D deficiency as a risk factor for obesity and T2D [55]. The decrease in BMI SDS concurs with earlier studies indicating that vitamin D repletion could potentiate weight loss and improve metabolic markers [47], [56], [57]. Vitamin D is believed to regulate adipocyte apoptosis leading to decreased fat mass [58], [59]. This vitamin D-associated weight loss may have contributed to the significant decrease in ALT concentrations in our T2D cohort.

### Vitamin D Deficiency and Alanine Transaminase

There was a significant decrease in ALT following vitamin D supplementation in patients with T2D compared to those with T1D. Several studies have reported an association between liver enzymes or calculated surrogate measures of NAFLD, such as fatty liver index, and the incidence of diabetes mellitus [13]–[16]. Recent studies suggest that NAFLD predicts prediabetes [12], and that the resolution of fatty liver could reduce the risk of incident diabetes mellitus [18]. We previously showed a strong inverse relationship between ALT and 25(OH)D in children and adolescents with T2D [22], while this present study reports a significant decrease in ALT levels in patients with T2D who received vitamin D supplementation for the management of hypovitaminosis D.

The mechanism of the transient, clinically-significant improvement in glycemic control in patients with T2D during the period of vitamin D supplementation is not clear. Vitamin D supplementation has been shown to improve β-cell function [23] and the augmentation of insulin synthesis and secretion once it is activated to its active form, 1,25-dihydroxyvitamin D, by 1α-hydroxylase enzyme which is expressed in β cells [60] [61]–[63]. This study however, suggests that an improvement in hepatic dysfunction, as shown by reduced ALT, could also be associated with an improved glycemic control.

One of the limitations of our study is its retrospective nature which could have introduced selection bias as only patients who fulfilled the inclusion and exclusion criteria were studied. We also did not directly evaluate subjects’ compliance with their vitamin D therapy. However, the post-treatment vitamin D levels were significantly elevated in patients with either T1D or T2D. Another limitation was that the vitamin D supplementation was not continued for longer than three months, and thus it was unclear whether the effect of vitamin D supplementation on glycemic control could have been sustained over a longer period of time. The lack of a relationship between ALT and 25(OH)D in T1D patients may be due to the fact that a fewer number of patients with T1D had ALT measured as compared to the larger proportion of patients with T2D who had ALT values. Specifically, forty two (47.7%) of the patients with T1D, and 36 (83.7%) of the subjects with T2D had ALT measured at baseline, while 12 (13.6%) of T1D and 29 (67.4%) of the T2D patients had ALT values following vitamin D supplementation. Another reason for a lack of an association between ALT and 25(OH)D could be due to the fact that subjects with T1D had normal mean serum ALT concentration at baseline compared to patients with T2D.

The strengths of this study stem from the fact that both the patients with either T1D or T2D received their care within the same clinical system, which is located in a similar geographical area around latitude 42° N, thus ensuring similar levels of insolation around the year. We had a relatively good sample size to enable us determine the differences in the various parameters studied. The availability of post-treatment 25(OH)D values confirmed that the subjects received the interventional therapy, while the availability of serial HbA1c values enabled us to establish a longitudinal model for the investigation of the duration of the glycemic effects of vitamin D supplementation on HbA1c and ALT in both T1D and T2D. Even though this is a retrospective study, it provides the much needed data on the effect of routine vitamin D supplementation on HbA1c, 25(OH)D, and ALT values in children and adolescents with either T1D or T2D. Such data will help establish the rationale for further investigation on the effects of vitamin D supplementation on these parameters in children and adolescents with diabetes mellitus.

## Conclusions

This study demonstrates that routine vitamin D supplementation in patients with diabetes mellitus is associated with a significant reduction in BMI SDS, ALT, and an initial, clinically-significant reduction in HbA1c in patients with T2D, but not T1D. The changes in these parameters were not associated with significant changes in the doses of either insulin or metformin. Even though these findings suggest that vitamin D deficiency could impair hepatic processes that promote β-cell function and glycemic control in patients with T2D; and that this hepatic dysfunction could be ameliorated by vitamin D supplementation with resultant improvement in glycemic control, our data are not adequate to confirm these conclusions. Further randomized controlled trials are warranted to determine the effect of vitamin D supplementation on glycemic control in T2D, and the optimal dose of adjunctive vitamin D therapy for the maintenance of long-term glycemic control in children and adolescents with diabetes mellitus.
